# Department of Gynecology

**Published:** 1894-07

**Authors:** 

**Affiliations:** F. R. C. S., Ed., Professor of Anatomy University of Texas; late Physician for Diseases of Women, Edinburgh Providence Dispensary; Galveston


### DEPARTMENT OF GYNECOLOGY.

EDITED BY WM. KEILLER, F. R. C. S., ED.,

Professor of Anatomy University of Texas; late Physician for Diseases of Women, Edinburgh Providence Dispensary.

BONE MARROW IN THE TREATMENT OF PERNICIOUS ANÆMIA. —Prof. T. R. Fraser (Edinburg) reports a case which gives new hope in the treatment of this deadly form of anæmia. The patient, male, aged 60, got worse rapidly under iron and arsenic, but improved promptly and steadily when three ounces daily of ox and calf bone marrow, uncooked, were administered. The case was of such urgency that at first the iron and arsenic were continued along with the marrow, and at other periods thirty grains daily of salol were given with the bone marrow (the iron and arsenic being stopped). Later on, however, the patient continued to improve under the marrow treatment only. The hæmocytes rose from 843,000 per cub. mill. to 3,900,000 per cub.

mill., and the hæmoglobin from 18 to 78% in a little over two months, and the patient was discharged fit for work. The case appears to justify the hope that bone marrow will be found to have a remedial value in some at least of the cases of pernicious anæmia.—*Brit. Med. Journal, June 2, '94.*

---

DR. A. SYKES-WARD reports a case where a piece of gum-elastic bogie 5½ inches long was found partly in the uterus, partly in the vagina, 11 months after it had been introduced (successfully) for the purpose of procuring abortion. The only result was a purulent vaginal discharge. It is remarkable that it should have been retained in utero after the miscarriage and that there should have resulted no peri-uterine inflammation.—*Brit. Med. Journal, June 2, '94.*

---

ACTION OF SALICYLIC ACID AND ITS COMPOUNDS ON THE UTERUS. H. N. Vineberg, M. D.—(*N. N. Medical Journal, June 23, 1894.*)

Given in full doses for sheumatism salicylic acid and salicylate of sodium have been found to cause abortion in the pregnant uterus, to increase the hemorrhage during the puerperium though not interfering with involution, and to induce a flow in patients who have suffered from scanty and irregular menstruation. Dr. Vineberg concludes from his observations:

1. Salicylic acid and its compounds may be found useful in scanty and delayed menstruation.

2. They should not be administered to pregnant women who have a predisposition to abort, or to women who suffer from menorrhagia or metrorrhagia.

3. Their administration should be watched carefully in all cases of pregnancy, and on the appearance of any "show" or anything resembling labor pains, they should be discontinued.

